# A qualitative study of online mental health information seeking behaviour by those with psychosis

**DOI:** 10.1186/s12888-016-0952-0

**Published:** 2016-07-11

**Authors:** Golnar Aref-Adib, Puffin O’Hanlon, Kate Fullarton, Nicola Morant, Andrew Sommerlad, Sonia Johnson, David Osborn

**Affiliations:** Division of Psychiatry, University College London, 149 Tottenham Court Road, London, W1T 7NF UK; Camden and Islington NHS Foundation Trust, 4th Floor, East Wing, St Pancras Hospital, 4 Saint Pancras Way, London, NW1 0PE UK

**Keywords:** Psychosis, Severe Mental Illness, Online, Internet, Health Information, Digital Technology

## Abstract

**Background:**

The Internet and mobile technology are changing the way people learn about and manage their illnesses. Little is known about online mental health information seeking behaviour by people with psychosis. This paper explores the nature, extent and consequences of online mental health information seeking behaviour by people with psychosis and investigates the acceptability of a mobile mental health application (app).

**Methods:**

Semi-structured interviews were carried out with people with psychosis (*n* = 22). Participants were purposively recruited through secondary care settings in London. The main topics discussed were participants’ current and historical use of online mental health information and technology. Interviews were audio-recorded, transcribed and analysed by a team of researchers using thematic analysis.

**Results:**

Mental health related Internet use was widespread. Eighteen people described searching the Internet to help them make sense of their psychotic experiences, and to read more information about their diagnosis, their prescribed psychiatric medication and its side-effects. Whilst some participants sought ‘expert’ online information from mental health clinicians and research journals, others described actively seeking first person perspectives. Eight participants used this information collaboratively with clinicians and spoke of the empowerment and independence the Internet offered them. However nine participants did not discuss their use of online mental health information with their clinicians for a number of reasons, including fear of undermining their clinician’s authority. For some of these people concerns over what they had read led them to discontinue their antipsychotic medication without discussion with their mental health team.

**Conclusions:**

People with psychosis use the Internet to acquire mental health related information. This can be a helpful source of supplementary information particularly for those who use it collaboratively with clinicians. When this information is not shared with their mental health team, it can affect patients’ health care decisions. A partnership approach to online health-information seeking is needed, with mental health clinicians encouraging patients to discuss information they have found online as part of a shared decision-making process. Our research suggests that those with psychosis have active digital lives and that the introduction of a mental health app into services would potentially be well received.

**Electronic supplementary material:**

The online version of this article (doi:10.1186/s12888-016-0952-0) contains supplementary material, which is available to authorized users.

## Background

Over 3 billion of the world’s population is now estimated to have access to the Internet (http://www.Internetworldstats.com/stats.htm). In the UK 83 % of adults have Internet access, and over half own a smartphone [1]. The Internet is changing the way people learn about and manage their illnesses. In a recent European survey, over 75 % of respondents felt that the Internet was a good resource for finding out more about health and 60 % reported using the Internet to look up health information [2]. Due to the increasing availability of the Internet and the anonymity it offers, online health resources may present an attractive source of information for those with stigmatised conditions [3]. It is reported that more than half of the people with first episode psychosis use the Internet as a source of information about their mental health [4] and that over 50 % of the people with psychiatric problems use the Internet to find out about their diagnosed mental health condition [5].

Current evidence suggests that people with psychosis access and use digital technology in a similar way to individuals unaffected by mental illness [6, 7]. However, little is known about how people with psychosis view and interact with mental health information online.

Although E-Mental health interventions are in their infancy, those that have been developed to support people with psychosis and their families have been well received by users [8, 9]. Online and mobile-phone based interventions are associated with improved medication management amongst people with psychosis and seem to be at least as effective as standard care in relation to adherence [8].

We aimed to explore the nature, extent and consequences of online health information seeking by people with psychosis, in order to inform future clinical practice and the potential development of a novel E-mental health app.

## Methods

### Settings

This study took place in Camden and Islington NHS Foundation Trust, which is a NHS mental health provider in an ethnically and socially diverse inner-London area. Participants were recruited through an Early Intervention Service for psychosis, a NHS residential Crisis House and an Acute Day Unit, which offers a day service to people in mental health crises. Ethical permission was obtained from a UK National Health Service Research Ethics Committee (REC reference: 13/EE/0222).

### Participants

Participants were purposively recruited to obtain views across age groups, gender, ethnicity, psychotic diagnoses, and educational levels.

Inclusion criteria:

- English-language speaker
- Aged 18–65 years
- Diagnosis of psychosis (schizophrenia, schizoaffective disorder, bipolar disorder with psychotic symptoms, persistent delusional disorder or psychosis not otherwise specified)
- Currently using or had previously used the Internet.

Eligible patients were initially identified and approached by a member of their mental health team. Those who expressed interest were contacted by the researcher, who provided further information and obtained informed consent. Taking account of the potential range of socio-demographic and clinical positions of our interviewees, we estimated that we would require between 15–25 participants to reach the point of theoretical saturation.

### Data collection

A semi-structured topic guide was used to ensure certain topics were discussed with every participant whilst remaining open enough to allow new areas to be explored depending on each participant’s views/experiences. One researcher (GA) retrieved and reviewed key papers about online mental health enquiry [5, 7, 10], identifying major themes for a draft topic guide. Other members of the research team reviewed the themes identified, and a pilot topic guide was agreed upon. This was piloted with 2 participants and was finalised in collaboration with the study research team. The same basic topic guide (See Additional file 1), with options to probe and explore answers, was used with all participants. The topic guide (see Additional file 1) covered:

- Participants’ current and historical health related Internet use
- The reported impact of this on their mental health
- Their current and historical use of E-Mental health technology such as mental health apps
- Their experience of, and attitudes towards, health related Internet use and E-Mental health technology

Semi-structured interviews, up to 90 min long, were conducted by GA, an Academic Clinical Fellow in Psychiatry, at the secondary care setting at which the participant was being seen. Demographic data (gender, age, ethnicity, education levels, psychosis and other co-morbid diagnoses) were also recorded.

### Analysis

The interviews were audio-recorded, transcribed verbatim, and any identifying information was removed to preserve anonymity. The manuscripts were imported to QSR NVivo 10 for Windows [11]. The material was analysed using thematic analysis [12]. To enhance validity, a second researcher (PO) separately coded ten of the transcripts and the developing coding frame was discussed and reviewed with the wider research team throughout analysis. Recruitment, data collection, and analysis occurred concurrently until saturation was reached. We judged this to have occurred after 22 participants’ transcripts were analysed, as no further themes were generated.

## Results

The characteristics of the participants are shown in Additional file 1: Table S1. The sample was predominantly from White and Black British backgrounds, single, and in receipt of employment or disability living allowance. Their ages ranged from 21 to 57. The most common diagnosis was Psychosis Not Otherwise Specified (Psychosis NOS). The duration of interviews varied from 21 to 65 min. One interview (P12) was cut short as the degree of psychotic symptoms experienced by the participant meant he was unable to complete the interview. Whilst data gathered from his initial responses are included in the results, he was unable to discuss the more complex questions regarding the extent and impact of his online mental health enquiry.

### Digital lives and overview

Eighteen of the participants reported that they had access to wireless Internet where they lived and most had a personal device from which they could access the Internet. Seventeen reported accessing the Internet daily, four others reported regular Internet use and only one person said they rarely used the Internet. Of the thirteen participants who owned smartphones, all described using general apps downloaded to their mobile devices, but none had used a mental health app and most stated that they had never heard of any.

Results are organised into four thematic sections.

1. How and why the majority of participants sought mental health information online.
2. Participants’ experiences in navigating, accessing, and processing this information.
3. Impact of online information on the participants’ emotions and behaviour and how this was influenced by their relationship with clinicians.
4. Respondents’ views on self-management apps for psychosis.

These key themes and findings are discussed below.

1. Seeking and Finding Mental Health Information Online

The majority of participants had used the Internet to find out more information about mental health (*n* = 18). Sixteen people described searching the Internet to help them make sense of their experiences (including delusions and other symptoms of psychosis), and read more information about their diagnosis, their prescribed psychiatric medication, and its side-effects. Three people discussed searching for mental health advocacy and mental health organisations. All of these participants reported using Google to direct them to relevant mental health information, with six participants stating that they often limit their browsing to the top Google search results. Wikipedia was the most frequently identified information source. One participant described not knowing the names of specific diagnoses, so he found that searching symptoms and then following links a useful way of navigating the Internet in search of mental health information. Two participants reported following links, one through a UK based mental health charity’s Twitter account and the other through the NHS Choices website. However, there was a general lack of awareness of NHS online resources or specific mental health charity websites and of how to access them, with only five participants reporting having had experience of using them. Despite feeling that NHS websites would be well informed and a responsible source of information, four participants discussed having never accessed them as they were unsure how to.

Information about medication and medication side-effects was the most common topic of mental health enquiry online (*n* = 15). Participants described going online to enhance their knowledge and understanding because the information they found was more detailed and in-depth than other resources, such as leaflets or clinician-provided information. Several participants described how accessing this detailed information helped them to feel better informed with regard to their medication and mental health problems, and how this led to a sense of security and reassurance. One person described how reading scientific explanations of her delusions helped her to manage her experiences.

*“I would have a difficult time not believing my delusions and it would help to look at a sort of medical thing [online] so I could affirm the idea that I was experiencing mental health problems.” (P04)*

Whilst some participants sought ‘expert’ online information originating from mental health clinicians and research journals, others described actively seeking first person perspectives. Participants described seeking these experiential accounts for a number of reasons. One described feeling resentful towards mental health services following his first compulsory admission to a mental health hospital and wanting to make sense of this by reading about other people’s experiences (P10). Some participants described looking for advice from people with lived experience on how to cope with their diagnosis or manage their mental health, while others simply wanted reassurance that they were not alone in their experiences of mental health problems. One participant reported that reading about other people’s experiences had helped her make sense of her own and another described how reading recovery stories gave him hope for his own future (P08).

“*Reading other people’s success stories regarding how they’ve gone back to a normal life can be, you know, somewhat reassuring.” (P08)*

A minority of participants had been advised to look up mental health resources online by family and mental health clinicians (*n* = 4). In contrast to participants who were internally motivated by a personal desire to further their understanding of mental health, these participants spoke of either looking only briefly, not having the motivation to fully explore the subject, or only looking at the recommended site if it was of specific interest to them.

*“My key worker at the hostel, she said she found out about it from somewhere … I didn’t go to the website yet. I just didn’t do it really, I had motivation difficulties really.” (P18)*

Four participants spoke of never having used the Internet for mental health related enquiry. These people all had Internet access at home and spoke of using it regularly for other purposes such as communication and social media. Whilst these participants were from a wide range of age groups and ethnic backgrounds, none of them had received higher education. When online mental health enquiry was discussed, one expressed an interest in accessing mental health information but reported that they had not thought of using the Internet to do so. The other three were aware that the Internet could be used for this purpose but either reported having no interest in further information about their mental health or treatment, or that they actively avoided information about mental health problems. All four described relying solely on written and verbal information provided by their mental health teams, and feeling satisfied that such information adequately met their needs. This seemed to preclude any felt need for independent enquiry.

*“Basically I don’t feel that it’s something I need to look up online. Me being in a mental health hospital, I put my trust in the doctors to come through for me to help me get better. If I was relying on it myself then I would look it up but because I am relying on it with other people, I don’t feel I need to look it up. I feel that they have the solutions.” (P06)*

2) Experiences of Mental Health Information Online

### Accessibility and availability

Many participants who used the Internet to search for mental health information described the benefits of having access to current and in-depth information online that was more accessible across space and time than other sources, including clinicians.

*“It’s readily available, it’s easily accessible. Not that a clinician isn’t, I mean … you can access it any time that you want … I suppose the accessibility is … really positive.” (P04)*

However, several participants described barriers to access that applied to all E-mental health information and services. Financial barriers were the most commonly cited. Several participants reported being unable to afford a replacement after their smartphones were broken, stolen or sold. Others were concerned about having sufficient data allowance on their mobiles to access E-resources and having to go a café to access public wireless Internet.

Four participants described the interplay of their mental health and Internet use. These participants reported reduced Internet use when they were unwell, due to either feeling unmotivated or finding Internet resources difficult to engage with. Conversely, two other participants described not wanting to access online mental health information or E-mental health services when they felt well. They explained that they did not have the time or did not want to think about their mental health difficulties.

*“I feel like I’m on top of my mental health at the moment. So yeah, I don’t feel any inclination to start planning out charts and stuff about how I’m doing … to me the focus on it is kind of what gets me down about it, you know? I prefer it if it just didn’t really exist.” (P10)*

One participant, who had a mild learning disability and a diagnosis of paranoid schizophrenia, spoke of needing support from a clinician to access online information as her symptoms and antipsychotic medication left her feeling too tired and unwell to navigate the Internet independently, beyond social media and online shopping. Others described lacking confidence with digital resources and felt they would need guidance or support from mental health clinicians before starting to use new online mental health resources.

### Navigation and comprehension

Participants’ experiences of finding and understanding information on the Internet varied. Many described the information as easier to understand than other written sources due to clearer presentation and simpler language.

*“I prefer Internet to books. Books [have] big words that I don’t even know the meanings. I prefer Internet, it’s easy words.” (P11)*

Several participants framed their ability to understand online information in terms of the level of professional expertise needed to comprehend it. Some described information they found on the Internet as accessible to the lay person, while others implied that finding and processing mental health information required specialist training and skill.

*“It’s very practical and … down to earth in how it’s written. You don’t have to be a mental health professional to get an understanding and benefit from it. So yea, it’s good.” (P8)*

However, not all participants spoke positively of the depth and abundance of information, and some described difficulties in accessing or processing it. Several participants described feeling overwhelmed by the large quantity of online information, or feeling incapable of understanding what they found. One participant felt that she lacked the medical expertise required to find and comprehend mental health information online (P17). Another preferred relying on clinicians to filter available information over independent research, having struggled with the amount and depth of online information when looking things up independently (P01).

*“I feel like I am fed up looking up things all the time … Just asking the doctors this time instead of looking it up … it’s not in depth … Not loads of pages. It’s a small summary of what’s available on Internet. The main bits not the whole lot.” (P01)*

### Reliability

There was a wide range of views with regard to the reliability of information on the Internet. Whilst some participants felt that it was accurate, others were more cautious about the nature of the information found online. Participants’ judgements of the reliability of online information were strongly linked to their level of trust in those to whom they credited authorship, and the qualifications of the author. Participants who thought that the information was accurate generally expressed a belief that trusted professionals, such as doctors or researchers, kept the information up-to-date. In particular, Wikipedia was identified as the most reliable source of health information online.

*“I look at my medications on Wikipedia and stuff like that … it has all the side-effects … really good researchers already scan all the books and have a big book list at the end of it.” (P18)*

Other participants were more cautious of the credibility of online health information. Some questioned its reliability due to having found contradictory information, and questioned the qualifications, motivations, and sources of those posting. Two participants expressed distrust towards the NHS and NHS professionals, and a consequent suspicion of information on NHS websites. They described anti-psychiatry and conspiracy theory websites as more reliable, and reported using these as their sole source of mental health information.

*“I kind of look at mental health and psychologists, I don’t trust them 100 %. So I never look up on anything that comes out of the NHS or it has to be conspiratorial if I look at mental health issues.”(P09)*

Four participants spontaneously expressed an interest in obtaining a recommended list of mental health websites. They either reported difficulty finding reliable sources despite spending hours looking through information online, or said that they did not know what was available. Two other participants said that the discussion from the interview had reminded them to search for sites beyond the top search results from Google.

### Anonymity and privacy

Over half of the participants who used online health resources spoke of the security and anonymity of the Internet. Participants described the importance of the privacy of this online world that could be accessed in their own space.

*“The Internet’s nature, you know, in itself it, there is a sense of security with it because you’re at home [and], I suppose, safe.” (P08)*

Participants also described feeling more relaxed when looking up information online than in a face-to-face meeting with a mental health clinician.

*“I suppose there can be a bit of pressure when you are speaking to the doctor because it can be a big deal to start on a new medication and it can be a nice to have a calmer look at the information.” (P04)*

3) The Patient-Internet-Clinician Relationship

*This section is based on data from seventeen people who used the Internet to access mental health information online. This does not include the four people who were non-users and the participant whose interview was terminated early and did not answer these questions.*

Participants who used the Internet for mental health information could be divided broadly into two groups. Eight people accessed Internet information, discussed this with their mental health team, and worked in conjunction with clinicians to make shared care decisions. For the purposes of the study we describe this group as *collaborators*. The other group, (*n* = 9), *parallel universes* were active users of the Internet. They used it to gather more information about their mental health, but did not bring this new information into consultations with their mental health clinicians. They kept their online searches, and the impacts of these, separate from discussions in clinical consultation, so that these two domains of knowledge and activity co-existed separately as parallel universes*.* Whilst both groups were derived from similarly diverse ethnic backgrounds, education levels and diagnoses, the participants who were collaborators were on average younger (30 vs. 38 years old), with fewer years’ contact with mental health services (3 vs. 10 years), fewer psychiatric admissions (3 vs. 7), and fewer involuntary admissions (1 vs. 2). The group of *collaborators* were all based at an EIS (Early Intervention in Psychosis) service. The *parallel universe* group were predominantly made up of residents of a Crisis House and/or an Acute Day Unit (*n* = 6) with a minority of EIS patients (*n* = 3). In this section we explore these distinct groups and the impact that these two styles of patient-internet-clinician relationship have on participants’ emotional experiences and clinical care.

### Fear and anxiety

Both groups of participants identified negative consequences of mental health enquiry online. The most commonly described experience was anxiety after reading about medication side-effects (*n* = 7). Four of these participants spoke of reading about sudden death in relation to antipsychotic medication, and another individual disclosed her fear of developing a learning difficulty after reading information suggesting that this happened to 1 % of those taking Aripiprazole (P11).

In the *parallel universe* group, two participants who had previously used the Internet as a source of information reported no longer doing so because of concerns about what they had read, explaining that they didn’t want to worry any more. Two other participants reported being so anxious about what they had read online that they had discontinued their medication without discussion with their clinicians. One said they had experienced a psychotic relapse and an admission to hospital as a result of stopping their medication (P5). The other described how finding this additional information online had led her to doubt the reliability of her mental health team (P11). Another individual described how reading about the side-effects of Olanzapine had made him want to take cocaine, which he felt would be safer.

*“I went online, and it worried me … I left my meds sitting there for a good three weeks, and I didn’t take them because I was scared about the side-effects. I got paranoid, let’s put it that way.” (P05, parallel universe)*

Those in the *collaborator* group expressed a similar underlying anxiety in relation to online information about the side-effects of antipsychotic medication. Unlike the *parallel universe* group, however, collaborators reported having discussed their concerns with their mental health teams. These participants described how clinicians had put frightening statistics into a clinical context, provided reassurance and, in some cases, proposed alternative medication. Whilst some described remaining anxious about possible medication side-effects, they reported that their medication adherence and behaviour remained unchanged following their Internet use.

Two participants in the *parallel universe* group reported feeling anxious and hopeless after reading or watching YouTube videos online about mental illness. Both described how this material left them feeling confused and unable to process the information. This was in contrast to the experiences of the *collaborator* group, where some individuals reported consulting a doctor in order to check the accuracy of the information they had found, which relieved the anxiety created by this supplementary information.

*“There’s a lot of information on the Internet that may not necessarily be correct. It may be correct at the time but it may have changed. So I was … checking and then going back to the doctors and asking.” (P13, collaborator)*

### Empowerment, control, and negotiating the clinician-patient relationship

For those in the *collaborator* group, both the act of independent research online and the understanding and knowledge gained as a result were closely linked with feelings of control and empowerment.

*“I think for one thing, it makes me feel more in control of things to be able to look at things independently and to get new information about it.” (P04, collaborator)*

*“I looked at my diagnosis … it’s very helpful, when you understand it. It sort of gives you the ability to recover from it.” (P2, collaborator)*

This greater sense of self-reliance did not seem to extend to those in the *parallel universe* group. These participants described their online mental health related enquiry as a solitary activity that they did not share with friends, family, or mental health professionals. Similarly, they reported healthcare-related decisions (for example, discontinuation of medication) that were influenced by online enquiry as having been made secretly or in private.

The majority of the *parallel universe* group explained their reluctance to share online information with clinicians in terms of their clinicians’ failure to initiate discussions about online health information or to recommend sites or E-mental health resources. However, participants’ explanations for their own reticence in clinical consultations regarding their online activity were suggestive of assumptions about power and knowledge in the clinician-patient relationship, in which the clinician was perceived as the ‘expert’ provider of information. Some participants expressed an explicit belief that doctors did not like patients exploring the Internet for supplementary information, but most described a more implicit sense that independent mental health information seeking online was somehow at odds with the status-quo:

*“It wasn’t that I felt I was usurping the medical authorities. It was just, I don’t know, it somehow felt like I shouldn’t be doing it. That sounds bizarre doesn’t it? Really. Because everyone is entitled to be self-informed.” (P20)*

*“I trust [my key worker] but it’s not really that I can tell [them] ‘oh I searched and this and I found out I am like schizophrenic people’ … I wouldn’t tell her that I think I have schizophrenia. It’s not really a nice thing to say. Because, well, she’s the person who finds my sicknesses, not me that finds it.” (P11)*

4) Future Use of Mental Health Apps

As part of the interview schedule, participants were asked about their experience and thoughts regarding mental health apps. None of the participants had used mental health apps and despite most using other apps in their daily life (*n* = 14), they said they were not aware that any mental health apps existed. They were asked about their views regarding the potential introduction of a self-management smartphone app and what would encourage or discourage them from using this.

There was a positive attitude towards the idea of a mental health self-management app, particularly among those who were currently completing a paper diary, with eighteen participants stating they would find it helpful. These participants felt an app should be clear, concise and easy to use. Some participants recommended tick boxes as an alternative to text input, while others suggested having free-text space to record any difficulties they had experienced. Several participants described how such an app could potentially help them overcome communication and recalled difficulties that they had experienced when seeing their mental health clinicians and discussing the nature and degree of psychotic or mood symptoms.

*“When I go to see my social worker or psychiatrist … it’s so hard sometimes to express yourself on how your mood has been, you don’t remember and you don’t know if there’s a pattern or not.” (P19)*

Five people spoke of an app giving them a sense of purpose and helping them to set goals for their recovery, with one participant likening the app to the acute day centre he was attending (P22).

*“A lot of the time when you’ve got a mental health problem you get up but you don’t know what to do with yourself … [an app] would be really useful because it would give them a goal and when you’ve got a goal, it helps.” (P20)*

Four participants spoke about the usefulness of having links or mental health information on the apps, which would allow them access to reliable and credible information through their mobile devices.

Perceived barriers to app use echoed those relating to general availability of the Internet, with financial barriers being commonly cited. Participants discussed being unable to afford smartphones, and two said that it would be advantageous to have an app that was also available on a tablet or computer. Others were concerned about having sufficient data allowance to allow access to such an app. Four participants described motivation to complete such an app as a key factor. Some felt that they may not be motivated to use it when unwell and others anticipated difficulty finding the motivation to engage with it on a daily basis, especially if they felt it was not useful. Two participants described lacking confidence with digital resources and felt they would need an induction or support from mental health clinicians before starting to use such an app. Several participants expressed a concern that digital health technology might replace human contact.

The four participants who said they would not use a self-management mental health app were from a range of age groups and ethnic backgrounds and included those from all three participant groups (*collaborators*, *parallel universe* and non-users of the Internet for mental health enquiry). Three out of four of these participants had smartphones and were using general apps. All of them stated that they felt stable with regards to their current mental health and felt that any additional emphasis on mental health or illness would be destabilising. The person who did not have a smartphone expressed concern about technology replacing the face-to-face contact with clinicians.

## Discussion

### The internet provides alternative perspectives and new information

People with psychosis are using the Internet to acquire mental health related information. Most of our participants use search engines and review the top search results only, which is typical of how the general population navigates the Internet for health information [10]. The popularity of Wikipedia, and the general lack of awareness of other sources of health information - such as NHS Choices - could be due to its high ranking on Google searches [13]. Alternatively it could be attributable to participants’ beliefs that it is the most comprehensive and up-to-date source on the Internet. There is a paucity of research on the credibility of health information on Wikipedia. One review suggests that although it has high accuracy, its readability is poor and does not meet the criteria for patient information leaflets and would benefit from further professional input [14].

Our research has shown that people affected by psychosis appreciate the accessibility of online health information and find this empowering. This is supported by existing evidence [7]. However, the results of this study suggest that while some participants find online information helpful and reassuring, for others who do not use this information collaboratively with their mental health team, it can lead to concern and affect health related decisions, including medication adherence. Since medication non-adherence is associated with a number of negative outcomes for people with psychosis [15], this is an important finding and warrants further research.

### Clinicians and patients need to communicate about the “virtual” world

Most participants did not discuss their use of online mental health information with their clinicians. These participants were generally older, had a longer psychiatric history with a greater number of compulsory admissions and were recruited from a non-EIS setting. These participants attributed this lack of discussion to the fact that their clinicians did not initiate the conversation. This may be because some mental health clinicians believe that patients, despite the growing body of evidence [6], are not using the Internet. Participants reported that they did not volunteer information about their Internet use for fear of undermining their clinician’s authority. This suggests a tension between the potential independence and empowerment offered by online health information seeking and the sense of dependence and respect for authority engendered by the traditional patient-clinician relationship. This has repercussions for shared decision-making beyond digital technology and may reflect perceived inequalities of power in the therapeutic relationship [16].

The participants who shared and discussed information with their clinicians were all from an EIS service. This may reflect the model of collaborative care that has been fostered and developed in these services as opposed to the more traditional hierarchal model of care. Previous qualitative research from EIS has highlighted the value that patients place on being involved in treatment decisions and working jointly with clinicians on their care plans [17]. Perhaps greater openness and equality in EIS therapeutic relationships has facilitated sharing and discussion regarding patients' online mental health searches.

A partnership approach to online health information-seeking is needed with mental health clinicians encouraging patients, particularly those with a longer psychiatric history and from an older age group, to discuss information they have found online as part of a shared decision-making process.

As other researchers have reported [7], patients want mental health clinicians to recommend websites and appropriate resources. This could provide an opportunity to initiate dialogue around patients’ mental health related Internet use. In addition, professionals could play an important role in enabling patients to critically evaluate and interpret information that they read on the Internet to reduce the risk of misinformation and alleviate concerns.

In other branches of medicine, E-health information is becoming increasingly embedded in the relationship with patients with speciality Wikipedia pages such as the Cancer Guidelines Wiki, created by the Australian Cancer Council (http://wiki.cancer.org.au/australia/Guidelines). Mental health clinicians should be advised and encouraged to follow suit.

### Limitations and strengths

The strengths of this study include the breadth of its sample, encompassing varying ethnicities, diagnoses, and levels of education. There is little previous relevant qualitative work and none of such depth.

Several limitations should be noted. Only participants who had used the Internet were recruited into the study and speaking of past Internet use introduces recall bias, so we may have over-estimated health related Internet use. While small samples are often sufficient to achieve theme saturation in qualitative research [18], a larger sample would increase confidence that our results reflected a full range of patients’ experiences.

All qualitative work is affected by the role of the interviewer. Since the interviewer has a clinical background, steps were taken to limit and critically appraise the influence of this on the results including involving non-clinicians in the research team and patients in our study design and analysis of the results.

### Clinical and research recommendations

Our findings are consistent with other studies suggesting that mental health mobile technology would be well received [19, 20]. It would require financial assistance for some patients, as lack of suitable funds was cited by a number of participants who did not have access to wireless Internet or smartphones. Mental health clinicians should consider ways to introduce and discuss online mental health enquiry in consultations with patients. This may alleviate concern about misinformation or overwhelming information and could have a positive impact on their health care decisions and outcomes.

## Conclusions

People with psychosis use the Internet to acquire mental health related information. This can be a helpful source of supplementary information particularly for those who use it collaboratively with clinicians. When this information is not shared with their mental health team, it can affect patients’ health care decisions. A partnership approach to online health-information seeking is needed, with mental health clinicians encouraging patients to discuss information they have found online as part of a shared decision-making process. Our research suggests that those with psychosis have active digital lives and that the introduction of a mental health app into services would potentially be well received.

## Abbreviations

Apps, an application, especially as downloaded by a user to a mobile device; EIS, Early Intervention in Psychosis ; NHS, National Health Service; Psychosis NOS, Psychosis Not Otherwise Specified.

## Additional file

Additional file 1: Topic Guide for Internet use amongst people who use mental health services. (DOCX 33 kb)
