# Supplementary material for: A qualitative study of online mental health information seeking behaviour by those with psychosis
Source: BMC Psychiatry. 2016 Jul 11;16:232. doi: 10.1186/s12888-016-0952-0 (PMC4940927; doi:10.1186/s12888-016-0952-0)
Supplement: Additional file 1: — Topic Guide for Internet use amongst people who use mental health services. (DOCX 33 kb) [file 12888_2016_952_MOESM1_ESM.docx]

**Additional File 1:**

**Topic Guide for Internet use amongst people who use mental health services**

| **1. Introduction (10 mins)**  *a) Nature and purpose of research*  *b)* *Anonymity of presentation of results*   - Introduce digital audio recorder - Stress confidentiality - Set ground rules   *c) Demographic data*  **2. Background (10 mins) Start recording**   - *Ask participant to say a little bit about their experiences of using the Internet for mental health related enquires*   **3. Current and historical use of the Internet (15 mins)**  a) How much do you currently use the Internet?  b) How and where do you access the Internet?  c) Do you have a personal smart phone, tablet, PC or laptop?  d) Where do you access the Internet?  e) Has your Internet use changed at all over the last 2 years? If yes- what has caused this change?  f) *Mental Health-* Do you access information about your mental health condition on the Internet?   - Do you access information about medication, diagnosis, mental health services, about your illness? Probe for any positive and negative aspects. - What sites do you favour for this? - Do you use mental health social media or online peer group support? Probe for any positive and negative aspects. - Do you have membership to forums related to mental health? Probe for any positive and negative aspects. - Have you ever purchased any medication online? - Does your Internet use change according to mental health? Have you noticed any relationship to your current mental health and your use of Internet? - Do your ever display care plans/medication/diagnosis on social media?   e) *Physical Health-* Do you access information about your physical health on the Internet?   - Do you access information about well-being on the Internet? Probe for any positive and negative aspects. - Can you name any sites you favour? - Do you use any physical health social media or online peer group support? Probe for any positive and negative aspects. - Do you have membership to any forums related to physical health? Probe for any positive and negative aspects. - Do you ever purchase health supplements online?   **4. Experiences of Internet (15 mins)- Now I would like us to think about the impact of the Internet on your mental health.**   - What has been your experience been in using the Internet? - Do you feel the Internet impacts on your well-being   Probe for positive and negative feelings. Probe further on:  a) Self esteem  b) Connectivity  c) Addiction/Distress  d) Impact on relationships  **5. Internet and mobile based resources (15 mins)**   - Are there any mental health based Internet resources or apps that you currently use to monitor your mental health? - Are there any physical health based Internet resources or apps that you currently use to monitor your physical health?   **a) Do you access any Internet based resources apps for relapse prevention and recovery planning?**  **b) Do you use the Internet for communication with key workers and psychiatrists?**  **FUTURE DEVELOPMENTS**  **Have you heard of any physical or mental Internet health resources including apps?**  Are there any Internet based mental health resources that you would find useful that may not be currently available?  Are there any Internet based physical health resources that you would find useful that may not be currently available?  **Probe further on views regarding:**  c) Online peer support group  d) Online therapy- individual and family  e) Self monitoring/carer monitoring apps  f) Physical health monitoring apps  g) Online psycho-education  **7. Conclude discussion (10 minutes)**  **Table S1: Baseline demographic and clinical characteristics of participants (n=22)** | | | |  |  |  |
| --- | --- | --- | --- | --- | --- | --- |
|  | **Characteristic** | | **N (%)** | | | |
| **Gender** | Female | | 12 (55) | | | |
|  | Male | | 10 (45) | | | |
| **Age (years)** | Median (range) | | 28 (21-57) | | | |
| **Ethnicity** | White UK | | 7 (32) | | | |
|  | White other | | 2 (9) | | | |
|  | Black British | | 4 (18) | | | |
|  | African/Caribbean | | 3 (14) | | | |
|  | Asian | | 1 (5) | | | |
|  | Other | | 5 (23) | | | |
| **Main diagnosis** | Psychosis Not Otherwise Specified | | 12 (55) | | | |
|  | Schizophrenia or Schizoaffective | | 5 (23) | | | |
|  | Bipolar Affective Disorder | | 3 (14) | | | |
|  | Other Psychotic Disorder | | 2 (9) | | | |
| **Past psychiatric hospitalization or psychiatric in-patient admission** | Yes | | 20 (91) | | | |
|  | No | | 2 (9) | | | |
| **Years of Contact with Mental Health Services** | | 1-5 | 14 (64) | | | |
|  |  | 6-10 | 2 (9) | | | |
|  |  | 10+ | 6 (27) | | | |
| **Highest level of Education** | No formal education | | 1 (5) | | | |
|  | Secondary | | 17 (77) | | | |
|  | University or College | | 4 (18) | | | |
| **Employment** | Full time | | 2 (9) | | | |
|  | Student | | 1 (5) | | | |
|  | No employment | | 19 (86) | | | |

**Table S2: Individual demographic information of participants**

| Participant | Age (years) | Gender | Diagnosis | Ethnicity | Marital Status | Service contact | Patient-Internet-clinician relationship* |
| --- | --- | --- | --- | --- | --- | --- | --- |
| P1 | 21 | F | BPAD with psychotic symptoms | Brazilian | Single | Acute Day Unit | Parallel Universe |
| P2 | 25 | M | Paranoid Schizophrenia | White British | Single | EIS & Acute Day Unit | Collaborator |
| P3 | 47 | F | Paranoid Schizophrenia | Mixed | Single | Acute Day Unit | Non user |
| P4 | 24 | F | Psychosis NOS | White British | Single | EIS | Collaborator |
| P5 | 25 | F | Psychosis NOS | Black British | Single | EIS | Parallel Universe |
| P6 | 22 | F | Psychosis NOS | Black British | Single | EIS | Non user |
| P7 | 25 | F | Psychosis NOS | Black African | Single | EIS & Acute Day Unit | Non user |
| P8 | 27 | M | Psychosis NOS | White British | Cohabiting | EIS | Parallel Universe |
| P9 | 31 | F | Delusional Disorder | White/Black Caribbean | Single | EIS | Collaborator |
| P10 | 26 | M | Psychosis NOS | Mixed | Single | EIS | Collaborator |
| P11 | 23 | F | Psychosis NOS | Iranian | Single | EIS | Parallel Universe |
| P12 | 29 | M | Psychosis NOS | White Other | Separated | EIS | *Interview suspended* |
| P13 | 40 | M | Psychosis NOS | Mixed | Partnership/Married | EIS | Collaborator |
| P14 | 23 | M | Psychosis NOS | White British | Single | EIS & Acute Day Unit | Collaborator |
| P15 | 42 | M | Psychosis NOS | Black African | Divorced | EIS | Non user |
| P16 | 22 | M | Psychosis NOS | British | Single | EIS | Collaborator |
| P17 | 50 | F | Delusional Disorder | Black British | Single | EIS | Collaborator |
| P18 | 43 | M | Paranoid Schizophrenia | White British | Single | Crisis House | Parallel Universe |
| P19 | 50 | F | BPAD with psychotic symptoms | White British | Partnership/Married | Crisis House | Parallel Universe |
| P20 | 57 | F | symptoms | White Irish | Partnership/Married | Crisis House & Acute Day Unit | Parallel Universe |
| P21 | 51 | F | Schizoaffective | Black British | Single | Crisis House | Parallel Universe |
| P22 | 47 | M | Schizoaffective | White British | Single | Acute Day Unit | Parallel Universe |

**Key: P1=Participant 1; BPAD = Bipolar affective disorder; NOS = Not otherwise specified**

***Please refer to Results- The Clinician-Patient-Internet Relationship section for further information and description of the groups.**
